# Digital inclusion finance, government subsidies and R&D investment—empirical evidence based on listed Chinese pharmaceutical firms

**DOI:** 10.3389/fpubh.2025.1545215

**Published:** 2025-04-09

**Authors:** XYv Zhao, Su Wang, Yuwen Chen

**Affiliations:** ^1^School of Business Administration, Shenyang Pharmaceutical University, Shenyang, China; ^2^Drug Regulatory Research Base of NMPA, Research Institute of Drug Regulatory Science, Shenyang Pharmaceutical University, Shenyang, China

**Keywords:** digital inclusive finance, R&D investment, traditional financial development level, pharmaceutical industry, moderating effect

## Abstract

Digital inclusive finance, with ‘universality’ as its core feature, can stimulate enterprises to increase capital investment in research and development (R&D) through diversified channels. Using the digital financial inclusion index of 337 prefecture-level cities in China and the empirical data of Shanghai and Shenzhen A-share-listed pharmaceutical manufacturing enterprises from 2011 to 2022, the article empirically examines the impact mechanism of digital financial inclusion on R&D investment of pharmaceutical manufacturing enterprises from the perspective of government subsidy by using a high-dimensional fixed-effects model, a two-stage systematic GMM, and a moderated-effects test method. It is found that digital financial inclusion has a positive incentive effect on pharmaceutical manufacturing enterprises to increase R&D investment, and this positive effect still holds after the endogeneity test and robustness test. The results of the moderating effect test indicate that government subsidies play a positive moderating role in the process of digital financial inclusion affecting enterprises’ R&D investment. Further analyses show that digital financial inclusion has a more significant role in promoting the intensity of R&D investment in pharmaceutical enterprises in private enterprises, small and medium-sized enterprises, and regions with lower levels of traditional financial development. Finally, policy suggestions are put forward according to the conclusion. Including the government to promote digital infrastructure and policy support, financial institutions to build data platforms to support pharmaceutical innovation, and enterprises to increase investment in research and development and expand financing channels to jointly promote the transformation and upgrading of the industry.

## 1 Introduction

Pharmaceutical manufacturing is an important industry related to national health, national security and social and economic progress. It is not only an indispensable part of the national economy, but also regarded as a top priority for strategic development by countries around the world (1). In China, with the steady rise in the income level of the population, the growing awareness of health care, the aging problem, and the deepening and improving reform of the medical system, the domestic residents’ demand for medicines is showing a continuously expanding trend. Against this background, China’s pharmaceutical manufacturing industry has ushered in an opportunity for rapid development and is playing an increasingly important role in the national economic system.

In recent years, with the continuous updating of information technology such as big data and blockchain, the integration of digital technology with the financial industry has been deepening. This trend has not only reshaped the mode of traditional financial services, but also broadened the boundaries of its services and significantly improved its service efficiency (2). Digital inclusive finance has changed the competitive situation of the traditional financial system and promoted the adjustment of financial structure and the optimization of financial efficiency by means of simplifying the distribution of financial functions and reducing the matching cost of supply and demand. Digital inclusive finance has a significant role in promoting enterprise innovation. On the one hand, digital inclusive finance helps enterprises scientifically plan their financial assets through six businesses, including payment and monetary funds. It can broaden financing channels and effectively reduce enterprises’ financial burdens in their daily production and operation. This enables enterprises to continuously conduct technological innovation research (3). On the other hand, the characteristics of inclusiveness have prompted digital inclusive finance to show stronger marginal innovation effects in small and medium-sized enterprises (4), private enterprises (5), enterprises with poor internal governance (6), and enterprises in regions with lagging financial development (7) and backward economic development (8).

The existing literature on digital inclusive finance mainly focuses on the impact of digital inclusive finance on corporate financing capabilities. During the period of COVID-19, the development of digital inclusive finance enhanced the availability of corporate financing, effectively alleviated the financing constraints faced by enterprises, and helped enterprises to carry out scientific and technological innovation (9). Jiang (10) found that digital inclusive finance has improved the innovation input and innovation output of enterprises, and this effect is more significant when enterprises have high financing efficiency and many investment opportunities. Jia and Liu (11) take the listed companies on the small and medium-sized board and the GEM from 2012 to 2018 as samples, and study how the development of digital finance affects enterprise innovation. It is found that the development of digital finance has a significant role in promoting the innovation of enterprises in the jurisdiction, and the R&D technology background of executives has a significant positive adjustment to this role. The political connection background of executives has an inhibitory effect on enterprise innovation. Peng et al. (12) established a fixed effect model and a mediating effect model based on the data of 30 provinces from 2012 to 2020, and empirically proved that financing constraints play a mediating role in the innovation process of digital economy-driven enterprises. Pang et al. (13) empirically proved that digital inclusive finance reduces the financing cost of private enterprises and promotes the high-quality development of private economy by alleviating financing constraints. Fusteret al. (14) found in the study of digital inclusive financial lending cases that financial institutions can approve corporate loan applications faster by using online services of digital inclusive finance.

In conclusion, digital finance has gradually become an important tool for promoting the upgrading and transformation of China’s economy and has been a research hotspot in academia in recent years. Scholars at home and abroad have conducted in-depth discussions on the various aspects of the impact of the role of the mechanism. From the macro level, digital finance has a facilitating effect on regional economic development, which can reduce urban–rural inequality and improve the well-being of residents. From the micro-level analysis, the development of digital finance can help alleviate the financing constraints of enterprises, optimize investment behavior, and improve the risk management of commercial banks. However, most of the existing studies cover all A-share listed companies or focus on start-ups and SME groups, and there is a relative lack of studies for the pharmaceutical manufacturing industry. It is worth noting that there are differences in the impact of digital financial inclusion on different enterprises, and the diversity of innovation subjects, including the nature of their property rights, their size, and the level of financial development of the region in which they are located, is not to be ignored. Therefore, it is of great significance to conduct in-depth research on the impact of digital inclusive finance on enterprises’ R&D investment and explore its mechanism for promoting enterprise innovation.

Based on this, this paper matches the data of digital inclusive finance in prefecture-level cities from 2011 to 2022 with the data of pharmaceutical manufacturing enterprises listed in Shanghai and Shenzhen A-shares, establishes a high-dimensional fixed effect model for empirical analysis, and deeply discusses the mechanism of digital inclusive finance to promote enterprises to increase R & D investment. In addition, the paper comprehensively explores diversity across ownership, size, and regional attributes. On this basis, it provides insights into how government subsidies play a moderating role between digital inclusive finance and corporate R&D investment. It provides strong theoretical support for China’s pharmaceutical industry to make full use of the inclusive characteristics of digital finance to enhance corporate innovation.

Compared with the results of existing studies, this study may have the following contributions: first, this paper adopts a moderating effects model to clarify the mechanism of digital inclusive finance on corporate R&D investment by identifying and testing the influence path of government subsidies. Second, there are fewer studies on digital inclusive finance for Chinese pharmaceutical companies. Focusing on the pharmaceutical industry with Chinese characteristics, this paper explores the impact of digital inclusive finance on enterprise R&D investment, which provides realistic references and lessons for the current innovation of the pharmaceutical manufacturing industry. Third, in terms of research content, this paper carries out a meticulous examination according to the dimensions of enterprise ownership characteristics, scale, regional attributes, etc., and explores the possible reasons behind it in light of the actual situation of China’s economic transformation and upgrading.

The structure of this study is as follows. The “Introduction” points out the research background of the topic and briefly outlines the main content and overall framework of the paper. In the section of “Theoretical Analysis and Hypothesis,” previous research results are systematically sorted out, and the hypotheses of this paper are put forward based on these theoretical and empirical foundations. In the section of “Research Design,” the data source, variable selection, and model setting of this study are elaborated in detail. The “Analysis of Empirical Results” section discusses the results. Finally, “Conclusions and Recommendations” and “Shortcomings and Prospects” are drawn.

## 2 Theoretical analysis and hypothesis

### 2.1 Digital inclusive finance and R&D investment

Pharmaceutical manufacturing enterprises are famous for their intensive R&D, and drug R&D has gone through many key links, such as laboratory development, drug toxicology research, clinical trials and new drug declaration (15). These stages require a large and unpredictable amount of upfront investment. However, internal funds are often difficult to meet the financial needs of innovation activities. Therefore, external financing has become an important source of corporate innovation funds (16). However, there are some problems in the traditional financial system, such as the information asymmetry of financial market, the lack of financial supply in underdeveloped areas and the high cost of enterprise financing (17), which constitute the financing constraints of enterprise innovation. When the enterprise R&D investment funds are insufficient, it is necessary to use the financial market to find financing to maintain R&D activities.

In this context, digital inclusive finance came into being. It alleviates the financing constraints of enterprises through various ways, and then encourages enterprises to increase R&D investment. First, digital inclusive finance reduces the threshold of financial services and broadens the financing channels of enterprises. Digital finance has broken away from the limitations of traditional financial services in terms of infrastructure and geographical distance, allowing enterprises to use diversified service models to broaden financing channels from multiple dimensions and reduce the financing pressure of innovative projects (18). Second, digital inclusive finance has effectively reduced financing costs, successfully prevented credit risks, and alleviated the problem of information asymmetry. Digital inclusive finance has established a perfect risk control system and information processing and monitoring system with modern information technology, which reduces the information asymmetry in traditional lending activities, gives full play to the role of information screening and risk screening (19), and reduces the financing cost of enterprises. Third, digital inclusive finance has also built a more robust corporate credit system, improved financing efficiency, and promoted the sound operation of enterprises. Under the new model, digital inclusive finance more comprehensively collects, excavates, collates and analyzes the financial data of enterprises, predicts the future development trend, and establishes a more perfect enterprise credit system (20).

In addition to the above direct effects, the development of digital inclusive finance has also had a direct technology spillover effect on other industries. As a product of the integration and innovation of finance and technology, it contains advanced technology and innovative ideas, and has the unique advantages of information sharing (21). This has promoted enterprises outside the financial industry to learn advanced experience and carry out technological transformation, forming a significant demonstration effect.

Finally, thanks to the strong support of the state, digital inclusive finance will benefit more enterprises and provide them with sustainable and affordable financial support (22). The enrichment of financial resources will intensify competition in the industry, thus prompting enterprises to continuously improve the level of technological innovation to ensure survival in the competition. Based on this, we propose hypothesis H1.

*H*1: Digital inclusive finance has a positive incentive effect on pharmaceutical manufacturing enterprises to increase R&D investment.

### 2.2 Digital inclusive finance, government subsidies and R&D investment

The theory of government intervention points out that the government plays an important macro-control role in the innovation R&D market, which can effectively curb the spread of risks (23). The positive externalities of enterprise innovation results are obvious, and the benefits obtained by enterprises from innovation results may be less than the benefits brought to society (24), which may lead to enterprises lacking innovation motivation and sticking to tradition. In order to stimulate the innovation power of enterprises, the government provides financial support for pharmaceutical manufacturing enterprises to innovate through subsidies and other means (25). This not only enhances the risk tolerance of enterprises, but also reduces the risk of innovation activities due to resource consumption. At the same time, the government subsidy funds do not need to be repaid by external financing, which provides a relatively loose trial and error environment for enterprises, and helps to promote enterprises to boldly try and innovate.

However, in the traditional financial system, the problem of information asymmetry between government and enterprises has always existed. It is difficult for the government to accurately evaluate the innovation projects of enterprises, which makes it difficult for many enterprises with innovation potential to obtain government subsidies (26). The development of digital finance has brought a turning point to this issue. With the extensive application and in-depth development of digital technology, the market environment has gradually become transparent. Big data technology has excavated and disclosed more enterprise information, so that the government can use modern information digital technology to conduct in-depth and comprehensive analysis of enterprise operations, form a precise portrait of the enterprise, and then correctly evaluate the development status and prospects of the enterprise, and more accurately determine the subsidy target (22). In addition, digital finance also enables the government to more effectively monitor the use and allocation of corporate funds. The government can carry out comprehensive supervision and management from the information before the engagement to the credit evaluation and communication after the engagement. This not only improves the pertinence and effectiveness of government subsidies, but also reduces the rent-seeking behavior and moral hazard of enterprises, and further improves the innovation performance of government subsidies (27). Based on this, we propose hypothesis H2.

*H*2: Government subsidies strengthen the positive incentive effect of digital inclusive finance on increasing corporate R&D investment.

## 3 Research design

### 3.1 Sample and data

The study selects China’s Shanghai and Shenzhen A-share listed pharmaceutical manufacturing enterprises as the research object, and the sample period is 2011–2022. Based on the availability and accuracy of the data, this paper follows the principles of previous scholars (28, 29) in dealing with the data of listed companies and excludes the companies that are labeled as ST or *ST during the study period. Companies with too many or incomplete missing values in the sample data are excluded, and the data of variables with few missing values are supplemented by using the interpolation method. A final sample of 1,939 observations from 252 listed companies was obtained. The data on pharmaceutical listed companies’ related indicators are obtained from the China Stock Market and Accounting Research Database (CSMAR)^1^ and Juchao Information Network.^2^ The data on economic and financial development at the regional level were obtained from the China Statistical Yearbook of the past years. The digital financial inclusion index is sourced from the “The Peking University Digital Financial Inclusion Index of China (PKU-DFIIC).” In order to avoid the negative impact of outliers on the model estimation, this paper uses Stata 16.0 statistical software to shrink the variables by 1% above and below the winsorize.

### 3.2 Variable selection and measurement

The main object of the study is the relationship between digital inclusive finance and innovation of pharmaceutical manufacturing enterprises. Therefore, R&D investment of pharmaceutical manufacturing enterprises is selected as the explained variable, the total index of digital inclusive finance as the explanatory variable, government subsidies as the mechanism variable, and other factors that may affect enterprise innovation as the control variable.

#### 3.2.1 Explained variable

The explained variable of the study is the R&D investment of listed pharmaceutical manufacturing enterprises. For the measurement of R&D investment, the existing literature mostly uses the ratio of R&D investment (R&D expenditure or R&D expenses) to the total assets (or operating income) of the enterprise (30), while ignoring the important role of R&D personnel in innovation output. On the one hand, it may be due to the serious lack of R&D personnel data. On the other hand, it may be due to the great uncertainty of the benefits brought by R&D personnel to the enterprise (31). To this end, this paper draws on the research ideas of Cheng et al. (32), and uses the ‘proportion of enterprise R&D investment to operating income’ in the main regression to measure the level of enterprise R&D investment. At the same time, in order to ensure the accuracy of the measurement results, in the robustness test, the practice of Wang et al. (31) is used to measure the R&D investment of enterprises by the interaction term of the natural logarithm of the R&D investment intensity and the number of R&D personnel, so as to examine the interaction between the two on innovation output.

#### 3.2.2 Explanatory variable

The core explanatory variable of the study is digital inclusive finance (index). Referring to the general practice of academia, it is measured by the Peking University Digital Inclusive Finance Index. This indicator covers three sub-dimensions: coverage breadth, depth of use, and degree of digital support services. It is the most authoritative and frequently used digital inclusive financial index in China. The time span is from 2011 to 2022, and the scope includes the total index of digital inclusive finance at the provincial, municipal and even some county levels. In order to further investigate the impact of digital inclusive finance on corporate R&D investment, this paper selects the total index of digital inclusive finance and its sub-indices in the prefecture-level cities where the sample enterprises are located to match the data of the sample enterprises, and discusses the impact of the three indicators on corporate R&D investment.

#### 3.2.3 Mechanism variable

The moderating variable used in this paper is government subsidies. Government subsidies refer to financial contributions and price or income support provided by a member’s government or public institution (33). The amount of government subsidies is derived from the current amount of government subsidies in the ‘non-operating income’ project disclosed in the company’s financial statements, and is measured by the proportion of government subsidies to operating income.

#### 3.2.4 Control variable

Based on the previous scholars’ research methods on the relationship between digital inclusive finance and enterprise innovation, this paper selects enterprise size (size), financial leverage (lev), fixed asset ratio (ppe), enterprise growth ability (growth), net asset profit rate (roa), equity concentration (share), and urban per capita GDP (lngdp) as control variables. The measurement and identification of the variables used for analysis are shown in Table 1.

**Table 1:** Variable names and identification.

Types	Name	Identification	Measurement method
Explained variable	R&D investment	intensity	Total R&D investment / year-end operating income
Explanatory variable	Digital inclusive finance	index	Peking University Digital Inclusive Finance Index
	Coverage breadth	breadth	
	Depth of use	depth	
	Digital support service degree	digitization	
Mechanism variable	Government subsidy	sub	Government subsidies / operating income
Control variables	Enterprise size	size	Natural logarithm of total assets
	Financial leverage	lev	Total liabilities / total assets at the end of the year
	Fixed asset ratio	ppe	Enterprise fixed assets at the end of the year / total assets at the end of the year
	Equity concentration	share	The number of shares held by the top 10 shareholders / the total number of shares
	Enterprise growth ability	growth	Operating income growth rate = (current year operating income-last year operating income) / last year operating income
	Net asset profit rate	roa	Net profit / total assets balance
	Urban per capita GDP	lngdp	The natural logarithm of urban per capita GDP

### 3.3 Regression model

#### 3.3.1 Benchmark regression model

The data used in this paper are annual unbalanced panel data. The Hausman test *p* value is less than 0.01, rejecting the null hypothesis, indicating that the sample data is suitable for the fixed effect model. Based on this, the following model is constructed to analyze the impact of digital inclusive finance on corporate R&D investment:


(1) $$\begin{array}{l} \mathrm{Intensit}y_{i,t}=\alpha +\mathrm{\beta inde}x_{c,t-1}+\sum \mathrm{\varphi Control}+\\ \sum \mathrm{year}+\sum \mathrm{province}+\varepsilon_{i,t} \end{array}$$

Intensity_i,t_ represents the R&D investment intensity of company i in year t. Index_c,t-1_ represents the prefecture-level city c in the t-1 year digital inclusive financial index. Control is a set of control variables, and εi,t is a random error term.

In the empirical process, considering that it takes a certain amount of time for digital inclusive finance to affect corporate R&D investment, this paper draws on the practices of Kang WG and others to lag the digital inclusive financial index, which can also appropriately mitigate the endogeneity problem of bidirectional causality (34). In the previous literature, many scholars have studied the effect of R&D inputs on the time lag of innovation output. Pang and Chen (35) found that on average, R&D investment contributes most to patent output 2 years after the investment, and the patent output of healthcare and biopharmaceuticals is positively correlated with the R&D investment in the first 4 years. Therefore, with reference to Su and Zhang (36, 39), model (Equation 2) is constructed to test the impact of lagged two-year digital financial inclusion on firms’ R&D investment.


(2) $$\begin{array}{l} \mathrm{Intensit}y_{i,t}=\alpha +\mathrm{\beta inde}x_{c,t-2}+\sum \mathrm{\varphi Control}+\\ \sum \mathrm{year}+\sum \mathrm{province}+\varepsilon_{i,t} \end{array}$$

Index_c,t-2_ represents the prefecture-level city c in the t-2 year digital inclusive financial index.

#### 3.3.2 Moderating effect model

In order to explore how government subsidies regulate the relationship between digital inclusive finance and corporate R&D investment, the model is constructed as follows:


(3) $$\begin{array}{l} \mathrm{Intensit}y_{i,t}=\alpha +\beta_{1}\mathrm{inde}x_{c,t-1}+\beta_{2}sub_{i,t}+\sum \mathrm{\varphi Control}+\\ \sum \mathrm{year}+\sum \mathrm{province}+\varepsilon_{i,t} \end{array}$$

(4) $$\begin{array}{l} \mathrm{Intensit}y_{i,t}=\alpha +\beta_{1}\mathrm{inde}x_{c,t-1}+\beta_{2}sub_{i,t}+\\ \beta_{3}\mathrm{inde}x_{c,t-1}\times sub_{i,t}+\sum \mathrm{\varphi Control}+\\ \sum \mathrm{year}+\sum \mathrm{province}+\varepsilon_{i,t} \end{array}$$

In Equation (3), $sub_{i,t}$ represents the government subsidy received by enterprise i in the t year. In Equation (4), $\mathrm{inde}x_{c,t-1}\times sub_{i,t}$ denotes the cross-multiplication of government subsidies and the digital inclusive financial index. The rest of the symbols mean the same as Equation (1).

### 3.4 Descriptive statistics

Table 2 reports the descriptive statistical results of the main variables. It can be found that the average value of R&D investment intensity is 6.744, and the standard deviation is 7.970, which indicates that there is a big difference in R&D investment level between pharmaceutical manufacturing enterprises. The average value of the digital inclusive financial index is 240.4, and the standard deviation is 73.27, which indicates that there are also great differences in the development level of digital inclusive finance among different cities.

**Table 2:** Descriptive statistics.

Variable	N	Mean	p50	sd	Min	Max
intensity	2,200	6.744	4.600	7.970	0.350	57.79
index	2,200	240.4	253.7	73.27	59.58	351.5
L.index	1939	230.2	243.1	71.44	38.37	391.9
L2.index	1,688	217.223	231.307	67.081	38.370	358.636
Size	2,200	21.89	21.82	0.978	19.72	24.31
lev	2,200	0.299	0.274	0.168	0.0400	0.752
ppe	2,200	0.212	0.190	0.111	0.0350	0.521
growth	2,200	0.190	0.125	0.471	−0.585	3.435
roa	2,200	0.0640	0.0610	0.0720	−0.204	0.301
lngdp	2,200	11.31	11.37	0.522	10.04	12.16
share	2,200	58.54	58.66	15.19	22.89	92.47

### 3.5 Correlation analysis

Before the regression of the variables, the Pearson correlation test is first performed. The calculation results are shown in Table 3. There is a correlation between the explanatory variables and the explained variables, and the correlation coefficient is basically less than 0.5. In addition, the VIF test of each variable shows that the VIF of each variable is less than 2. Therefore, it can be considered that there is no multicollinearity between the variables. In addition, the coefficient between the variable index and intensity is 0.301, and it is significant at the level of 1%, which preliminarily proves the positive correlation between digital inclusive finance and R&D investment.

**Table 3:** Correlation analysis.

Variable	Intensity	Index	Size	lev	ppe	Growth	roa	Share	lngdp
intensity	1								
index	0.301***	1							
size	−0.052**	0.163***	1						
lev	−0.112***	−0.056**	0.239***	1					
ppe	−0.114***	−0.071***	−0.087***	0.259***	1				
growth	0.166***	−0.0330	−0.0330	−0.051**	−0.105***	1			
roa	−0.215***	−0.061***	0.0280	−0.387***	−0.225***	0.187***	1		
share	0.114***	0.00600	−0.068***	−0.250***	−0.117***	0.114***	0.235***	1	
lngdp	0.282***	0.562***	0.100***	−0.097***	−0.127***	0.042**	0.00300	0.134***	1

*, **, *** indicate significant at the 10 percent, 5 percent, and 1 percent levels, respectively.

## 4 Analysis of empirical results

### 4.1 Benchmark regression estimation

In this paper, models (1) and (2) are subjected to multivariate unbalanced panel data regression analysis, and the results are shown in Table 4. Columns (1) and (2) examine the relationship between the digital inclusive financial index of the city where the enterprise is located and the R%D investment of the enterprise, wherein (1) controls the year and the urban fixed effect, and (2) adds control variables on the basis of column (1). Similarly, columns (3) and (4) test the relationship between the lagged two-year digital financial inclusion index and firms’ R&D investment in the city where firms are located, with column (3) controlling for year and city fixed effects, and column (4) adding control variables to column (3). The estimated coefficient of both lagged one and lagged two digital inclusive finance index are positive at the 1% significance level, indicating that the development of digital inclusive finance has a significant role in promoting the increase of R&D investment in pharmaceutical manufacturing enterprises. When considering the impact of control variables on corporate R&D investment at the same time, the total digital financial index still has a positive impact on corporate R&D investment, and with the addition of enterprise-related control variables, this positive impact is more obvious (at this point the coefficients are 0.074 and 0.068, respectively). The regression coefficients of the lagged one-period digital financial inclusion index and current enterprise R&D investment are larger than those of the lagged two periods, and the model fit R^2^ is also higher (0.256 > 0.227). This indicates that the model fit superiority in the lagged period is better; there is a lag in the enhancement effect of digital financial inclusion on the R&D investment of listed companies in China’s pharmaceutical manufacturing industry, and the lagged effect is then better reflected in the first period.

**Table 4:** Benchmark regression estimation.

	(1)	(2)	(3)	(4)
Variable	Intensity	Intensity	Intensity	Intensity
L.index	0.067^***^	0.074^***^		
	[0.011]	[0.018]		
L2.index			0.060^***^	0.068^***^
			[0.012]	[0.020]
size		−0.665^***^		−0.681^***^
		[0.215]		[0.223]
lev		−6.083^***^		−5.413^***^
		[1.281]		[1.222]
ppe		−6.138^***^		−5.447^***^
		[1.651]		[1.746]
growth		2.484^***^		1.336
		[0.824]		[0.902]
roa		−33.576^***^		−27.641^***^
		[5.486]		[5.766]
share		0.060^***^		0.058^***^
		[0.012]		[0.013]
lngdp		−0.611		−0.519
		[0.612]		[0.668]
_cons	−8.732^***^	12.613^**^	−6.374^**^	13.962^**^
	[2.565]	[6.180]	[2.599]	[6.455]
*N*	1939	1939	1,688	1,688
R^2^	0.158	0.256	0.156	0.227

Standard errors in brackets. ^*^p < 0.1, ^**^p < 0.05, ^***^p < 0.01.

The results of Table 4 show that there is a significant positive impact between the digital inclusive financial index and the R&D investment of pharmaceutical manufacturing enterprises. Digital finance has significantly broadened the financing channels of enterprises by lowering the threshold of financial services. This change enables pharmaceutical manufacturing enterprises to get rid of the limitations of traditional financial services in infrastructure and geographical distance, and broaden the financing path from multiple dimensions with the help of diversified service models, thus effectively reducing the financing pressure of innovative projects and increasing the capital investment in R&D innovation. In addition, by reducing information collection, processing, risk assessment and transaction costs, digital inclusive finance further reduces the financing costs of pharmaceutical manufacturing enterprises and provides more solid financial support for enterprise R&D. Based on the above research, the previously proposed hypothesis H1 is verified.

### 4.2 Regression analysis of the sub-indicators

Previous studies have concluded that the overall indicators of digital inclusive finance help pharmaceutical manufacturing enterprises to increase R&D investment, that is, with the overall development of digital inclusive finance, the higher the technological innovation ability of pharmaceutical manufacturing enterprises. In the above correlation analysis, it can be seen that the three sub-indicators of digital inclusive finance have a positive impact on corporate R&D investment. In the benchmark regression, the three sub-indicators are further tested. As shown in Table 5, the depth of use, breadth of coverage and degree of digital support services of digital inclusive finance have a positive contribution to the increase of R&D investment of pharmaceutical manufacturing enterprises.

**Table 5:** Sub-indicators regression results.

	(1)	(2)	(3)
Variable	Intensity	Intensity	Intensity
L.breadth	0.043^***^		
	[0.014]		
L.depth		0.052^***^	
		[0.015]	
L.digitization			0.032^***^
			[0.009]
size	−0.667^***^	−0.628^***^	−0.612^***^
	[0.216]	[0.215]	[0.214]
lev	−6.335^***^	−6.287^***^	−6.540^***^
	[1.291]	[1.282]	[1.282]
ppe	−5.976^***^	−5.731^***^	−5.576^***^
	[1.656]	[1.623]	[1.612]
growth	2.543^***^	2.454^***^	2.498^***^
	[0.831]	[0.827]	[0.827]
roa	−33.934^***^	−33.154^***^	−33.594^***^
	[5.520]	[5.485]	[5.490]
share	0.060^***^	0.058^***^	0.057^***^
	[0.012]	[0.012]	[0.012]
lngdp	−0.229	0.779^*^	1.565^***^
	[0.662]	[0.437]	[0.373]
_cons	15.731^**^	1.280	−3.743
	[7.130]	[5.844]	[6.211]
*N*	1939	1939	1939
R^2^	0.253	0.255	0.254

Standard errors in brackets. ^*^p < 0.1, ^**^p < 0.05, ^***^p < 0.01.

By comparing the regression coefficients of the three models, it can be seen that the depth of use has the most significant impact on pharmaceutical manufacturing enterprises, followed by the breadth of coverage and the degree of digital support services, indicating that for pharmaceutical manufacturing enterprises, continuing to strengthen the depth of use is more likely to promote the innovation of pharmaceutical manufacturing enterprises. The depth of use reflects the degree of in-depth application of digital inclusive financial tools and services by enterprises. For pharmaceutical manufacturing enterprises, the in-depth use of digital inclusive finance can bring more financial support, risk management tools and market information. Deepening the use of digital inclusive finance can also help enterprises optimize production processes, improve production efficiency, and thus enhance overall competitiveness. Compared with the two indicators of digital inclusive financial coverage breadth and digital support service degree, the coverage breadth has a greater impact on pharmaceutical manufacturing enterprises. Coverage breadth refers to the number or scope of pharmaceutical manufacturing enterprises that digital inclusive finance can reach. Although the increase in coverage breadth means that more enterprises have access to digital inclusive financial services, simply increasing the coverage breadth is not enough to ensure that these services can truly be transformed into the technological innovation power of enterprises. Therefore, while expanding the coverage, it is also necessary to focus on the improvement of service quality and the precise matching of services to ensure that digital inclusive finance can truly benefit enterprises in need. The degree of digital support services reflects the quality of technical support, consulting and training services provided by the digital inclusive amount platform for enterprises. Although these services have a positive effect on improving enterprises’ digital capabilities and innovation capabilities, their impact may be relatively weak compared to the depth of use and breadth of coverage. However, with the in-depth application of digital inclusive finance by enterprises, the importance of digital support services may gradually become prominent. Therefore, the digital inclusive financial platform should continuously improve the quality of service to meet the growing digital needs of enterprises.

### 4.3 Robustness test and endogeneity discussion

In order to ensure the robustness of the regression, in addition to the above lag variable method test, this paper uses three methods to test the robustness of the basic model, which are to replace the explained variables, shorten the sample period interval, and eliminate some factors.

#### 4.3.1 Replace the explained variable

To test the robustness of the benchmark regression results, this paper replaces the measure of R&D investment in the basic regression with the interaction term between the intensity of R&D investment and the natural logarithm of the number of R&D personnel. The robustness test results of the substitution variables in Table 6 show that the coefficients of the total indicators and the three sub-dimension indicators of digital inclusive finance are still significantly positive, indicating that digital inclusive finance can show a significant positive effect on the R&D investment of pharmaceutical enterprises, which further verifies the relevant assumptions proposed in this paper.

**Table 6:** Replace the explained variable regression results.

	(1)	(2)	(3)	(4)	(5)
Variable	Intensity	Intensity	Intensity	Intensity	Intensity
L.index	34.276^***^	41.555^***^			
	[7.341]	[10.306]			
L.breadth			27.477^***^		
			[8.154]		
L.depth				28.496^***^	
				[8.566]	
L.digitization					13.569^***^
					[4.847]
size		1918.470^***^	1913.357^***^	1939.472^***^	1947.433^***^
		[169.404]	[168.902]	[170.673]	[170.142]
lev		−1.9e+03^**^	−2.0e+03^***^	−2.0e+03^***^	−2.2e+03^***^
		[753.431]	[753.052]	[765.598]	[758.998]
ppe		−501.014	−456.347	−266.837	−163.980
		[900.209]	[905.969]	[880.396]	[877.239]
growth		971.220^**^	1003.638^**^	955.776^**^	985.762^**^
		[441.183]	[443.626]	[444.433]	[444.405]
roa		−8.3e+03^***^	−8.5e+03^***^	−8.1e+03^***^	−8.3e+03^***^
		[2976.659]	[2991.785]	[2978.104]	[2988.810]
share		32.629^***^	32.592^***^	31.504^***^	30.887^***^
		[6.481]	[6.467]	[6.486]	[6.479]
lngdp		−845.893^**^	−796.798^*^	−47.452	405.309
		[375.141]	[433.740]	[274.154]	[251.810]
_cons	−5.4e+03^***^	−4.0e+04^***^	−3.8e+04^***^	−4.7e+04^***^	−4.9e+04^***^
	[1677.658]	[3599.310]	[4120.096]	[3633.189]	[3876.347]
*N*	1939	1939	1939	1939	1939
R^2^	0.137	0.265	0.263	0.264	0.261

Standard errors in brackets. ^*^p < 0.1, ^**^p < 0.05, ^***^p < 0.01.

#### 4.3.2 Shorten the sample period interval

The development degree of enterprise innovation activities and digital inclusive finance is closely related to the financial environment of the whole country and even the whole world. If the impact of these external factors is ignored, it may lead to a certain degree of deviation in the regression analysis results. In the time dimension of this paper, the impact of the 2015 Chinese stock market crash, a major financial event, cannot be ignored. However, from a practical point of view, it is difficult to accurately measure and evaluate the impact of such sudden financial events through certain specific variables. Therefore, to ensure the accuracy and reliability of the research, this paper excludes the impact of the 2015 Chinese stock market crash. In addition, considering the more rapid development of China’s digital inclusive finance after 2016, this paper adjusts the sample interval of the study to 2016–2022. Table 7 reports the regression results, and the research conclusions are still robust.

**Table 7:** Replacement sample period interval regression results.

	(1)	(2)	(3)	(4)	(5)
Variable	Intensity	Intensity	Intensity	Intensity	Intensity
L.index	0.075^***^	0.080^***^			
	[0.013]	[0.021]			
L.breadth			0.049^***^		
			[0.016]		
L.depth				0.063^***^	
				[0.019]	
L.digitization					0.038^***^
					[0.011]
size		−0.710^***^	−0.703^***^	−0.671^**^	−0.655^**^
		[0.265]	[0.266]	[0.265]	[0.264]
lev		−7.355^***^	−7.610^***^	−7.759^***^	−7.977^***^
		[1.749]	[1.763]	[1.746]	[1.746]
ppe		−7.795^***^	−7.605^***^	−7.344^***^	−7.103^***^
		[2.084]	[2.092]	[2.054]	[2.027]
growth		3.183^***^	3.247^***^	3.157^***^	3.169^***^
		[0.937]	[0.944]	[0.938]	[0.942]
roa		−41.157^***^	−41.392^***^	−41.013^***^	−41.123^***^
		[6.246]	[6.274]	[6.246]	[6.249]
share		0.081^***^	0.080^***^	0.079^***^	0.078^***^
		[0.016]	[0.016]	[0.016]	[0.016]
lngdp		−0.787	−0.443	0.680	1.691^***^
		[0.810]	[0.879]	[0.625]	[0.514]
_cons	−12.144^***^	12.162	16.625^*^	−0.494	−6.794
	[3.448]	[8.081]	[9.314]	[7.618]	[8.088]
*N*	1,459	1,459	1,459	1,459	1,459
R^2^	0.143	0.267	0.263	0.266	0.264

Standard errors in brackets. ^*^p < 0.1, ^**^p < 0.05, ^***^p < 0.01.

#### 4.3.3 Excluding some factors

Because of its unique position, China’s municipalities show obvious particularity in economic development, central policy tilt and other aspects compared with other prefecture-level cities. They have stronger policy independence, more prosperous economic development environment and more prominent resource absorption capacity. These characteristics may lead to significant differences in the development of digital inclusive finance and technological innovation between municipalities and general prefecture-level cities. To ensure the accuracy and pertinence of the research, this paper deletes the samples of municipalities directly under the central government to conduct regression again, and Table 8 reports the regression results. The conclusion of the study did not change significantly.

**Table 8:** Excluding some factors affecting the regression results.

	(1)	(2)	(3)	(4)	(5)
Variable	Intensity	Intensity	Intensity	Intensity	Intensity
L.index	0.063^***^	0.072^***^			
	[0.012]	[0.019]			
L.breadth			0.050^***^		
			[0.013]		
L.depth				0.050^***^	
				[0.016]	
L.digitization					0.027^***^
					[0.009]
size		−0.350^*^	−0.366^**^	−0.302^*^	−0.289
		[0.179]	[0.179]	[0.179]	[0.177]
lev		−4.596^***^	−4.721^***^	−4.925^***^	−5.207^***^
		[1.292]	[1.296]	[1.307]	[1.310]
ppe		−4.335^**^	−4.294^**^	−3.840^**^	−3.674^**^
		[1.829]	[1.836]	[1.786]	[1.775]
growth		2.073^**^	2.128^**^	2.059^**^	2.127^**^
		[1.039]	[1.047]	[1.043]	[1.045]
roa		−22.727^***^	−22.903^***^	−22.436^***^	−22.897^***^
		[6.086]	[6.118]	[6.094]	[6.112]
share		0.043^***^	0.042^***^	0.041^***^	0.039^***^
		[0.013]	[0.013]	[0.013]	[0.013]
lngdp		−0.633	−0.663	0.778^*^	1.551^***^
		[0.602]	[0.637]	[0.435]	[0.361]
_cons	−7.784^***^	5.962	11.941^*^	−5.501	−10.084^*^
	[2.552]	[5.394]	[6.455]	[4.909]	[5.429]
*N*	1,536	1,536	1,536	1,536	1,536
R^2^	0.161	0.215	0.212	0.212	0.209

Standard errors in brackets. ^*^p < 0.1, ^**^p < 0.05, ^***^p < 0.01.

#### 4.3.4 Endogenous discussion

In this paper, a two-stage system GMM is used to solve the endogenous problem of the model. The instrumental variable uses the average spherical distance from each city to Hangzhou (37) and the Internet penetration rate of each city (38). The instrumental variable is used as the first-order lag term of the explanatory variable to estimate the regression of the model, and the AR (2) and Hansen values are used to test the validity of the model. AR (1) and AR (2) show that the first-order difference has autocorrelation, and the second-order difference does not have autocorrelation, so the GMM estimation is effective. The Hansen J test statistic is not significant, indicating that the model does not suffer from over-identification, the instrumental variables are valid, and the model set-up is reasonable. The results of Table 9 show that after considering the possible endogenous problems, the promotion effect of digital inclusive finance on the increase of R&D investment of pharmaceutical manufacturing enterprises is still established, and the regression results are positively correlated at the 5% significance level.

**Table 9:** Two-stage system GMM regression results.

	(1)
Variable	Intensity
L.intensity	0.931***
	(0.038)
L.index	0.046**
	(0.021)
size	0.149
	(0.214)
lev	−1.567
	(1.498)
ppe	−5.360***
	(1.794)
growth	−0.751
	(0.730)
roa	−2.300
	(4.076)
share	0.023*
	(0.013)
lngdp	−2.056
	(1.398)
Constant	7.399
	(12.794)
Number of id	252
Ar(1)	0.000182
Ar(2)	0.141
Hansen	0.247
N	1939

Standard errors in brackets. ^*^p < 0.1, ^**^p < 0.05, ^***^p < 0.01.

### 4.4 Mechanism analysis

Table 10 shows that the interaction coefficient between digital inclusive finance and government subsidies (L.index × sub) is significantly positive at the level of 1%, indicating that the acquisition of government subsidies enhances the role of digital inclusive finance in promoting R&D investment of enterprises. Hypothesis 2 is supported. The main reason is that government subsidies not only directly alleviate the financial pressure faced by enterprises due to innovative R&D and meet their capital needs, more importantly, it also sends a positive signal to social investors, indicating that these enterprises and their innovative projects have high quality and potential. This signal effect enhances the recognition and financing intention of the capital market for innovative R&D projects, and promotes a more balanced and efficient flow of financial resources between the supply and demand sides of innovative projects, thus continuously injecting new vitality and motivation into corporate innovation activities.

**Table 10:** Test results of moderating effect.

	(1)
Variable	Intensity
L.index	0.075^***^
	[0.017]
sub	−1.066
	[0.820]
L.index×sub	0.019^***^
	[0.004]
size	−0.007
	[0.180]
lev	−5.974^***^
	[1.076]
ppe	−3.481^***^
	[1.316]
growth	2.047^***^
	[0.573]
roa	−22.387^***^
	[3.914]
share	0.035^***^
	[0.010]
lngdp	−0.800
	[0.544]
_cons	0.198
	[4.926]
*N*	1939
R^2^	0.419

Standard errors in brackets. ^*^p < 0.1, ^**^p < 0.05, ^***^p < 0.01.

### 4.5 Heterogeneity analysis

#### 4.5.1 Equity nature

Due to the heterogeneous impact of equity attributes on business behavior and external financing environment of enterprises, to explore the potential differential effects of the development of digital inclusive finance on enterprises of different ownerships, the sample is divided into state-owned enterprises and private enterprises according to the equity attributes of enterprises. Columns (1) and (2) in Table 11 show the sample regression results. The results show that digital inclusive finance can significantly enhance the technological innovation ability of private enterprises, but has no significant impact on state-owned enterprises. The reason may be that private enterprises often face financing difficulties due to the limitations of the traditional financial system, but the rise of digital inclusive finance has broken this deadlock. By reducing the information asymmetry between banks and enterprises, optimizing the allocation of financial resources, it provides more financing opportunities for private enterprises. This not only alleviates the financial pressure of enterprises, but also injects a steady stream of power into the technological innovation of enterprises. In contrast, the situation of state-owned enterprises is different. Although digital inclusive finance also brings them the convenience of financing, because the business objectives of state-owned enterprises are not only to maximize benefits, but also to shoulder social responsibilities, they have not shown the same enthusiasm as private enterprises in technological innovation. In addition, there are differences between state-owned enterprises and private enterprises in the executive incentive system, which also leads to their differences in technological innovation willingness. To clarify whether there is a significant difference between the two sub-samples after grouping, this paper uses Chow Test to test the regression coefficients of sub-samples of state-owned enterprises and private enterprises. The calculation results show that there are significant differences between the two sub-samples, so sample grouping analysis can be carried out according to equity heterogeneity.

**Table 11:** Heterogeneity analysis.

	(1)	(2)	(3)	(4)	(5)	(6)
Variable	Private	State-owned	Lower	Higher	Small and medium	Large
L.index	0.081^***^	0.044^**^	0.069^***^	0.059	0.089^***^	0.041^*^
	[0.021]	[0.020]	[0.018]	[0.047]	[0.024]	[0.023]
size	−0.194	−0.604^**^	−0.396^*^	−1.098^***^	−0.702^*^	0.037
	[0.245]	[0.247]	[0.203]	[0.409]	[0.403]	[0.369]
lev	−6.162^***^	−5.502^***^	−5.040^***^	−7.168^***^	−3.308^**^	−10.444^***^
	[1.611]	[1.639]	[1.221]	[2.597]	[1.527]	[2.577]
ppe	−4.806^**^	−1.553	−3.243	−9.226^***^	−7.145^***^	−5.389^***^
	[1.997]	[1.556]	[2.098]	[2.667]	[2.028]	[2.035]
growth	2.643^***^	−0.444	1.224	3.527^***^	3.558^***^	0.230
	[0.903]	[0.405]	[1.003]	[1.161]	[1.053]	[0.684]
roa	−39.135^***^	−5.403^*^	−21.490^***^	−48.052^***^	−38.466^***^	−19.897^**^
	[6.019]	[3.217]	[7.161]	[8.565]	[6.725]	[8.347]
share	0.100^***^	−0.031^**^	0.023^*^	0.115^***^	0.070^***^	0.019
	[0.015]	[0.014]	[0.013]	[0.022]	[0.017]	[0.017]
lngdp	0.318	−4.067^***^	−0.973	2.724^*^	−0.451	0.361
	[0.650]	[0.856]	[0.651]	[1.566]	[0.766]	[0.845]
_cons	−11.876^*^	58.470^***^	12.990^**^	−14.725	7.264	−3.864
	[6.816]	[9.825]	[6.122]	[14.823]	[9.780]	[10.192]
Chow	29.42***		6.66***		9.84***	
*N*	1,483	456	1,200	738	1,312	627
R^2^	0.301	0.418	0.230	0.308	0.319	0.363

Standard errors in brackets. ^*^p < 0.1, ^**^p < 0.05, ^***^p < 0.01.

#### 4.5.2 Regional heterogeneity

When we discuss the impact of digital inclusive finance on corporate R&D investment, we must also consider the key factor of regional traditional financial development level. Columns (3) and (4) in Table 11 examine the different effects of digital inclusive finance on R&D investment of pharmaceutical manufacturing enterprises in regions with low and high levels of traditional financial development. The results show that, on the whole, digital inclusive finance has significantly increased the R&D investment of enterprises, and the Chow test of the coefficient of difference between groups is significant. In areas with low levels of traditional financial development, enterprises are facing more severe financing challenges. Due to the lack of service coverage and high financing threshold of traditional financial institutions, it is often difficult for enterprises in these areas to obtain sufficient financial support. However, the rise of digital inclusive finance has brought new hope to these enterprises. It uses digital technology to reduce the threshold and cost of financial services, making it easier for companies to obtain financing support. Therefore, digital inclusive finance has played an important role in improving the shortage of traditional finance and helping the rational allocation of financial resources. Especially in areas with low levels of traditional financial development, this positive impact is more profound and significant.

#### 4.5.3 Scale heterogeneity

Enterprise scale is a key indicator to measure enterprise resources and strength. Enterprises of different sizes have different social resources, market competitiveness and profitability. Therefore, the impact of digital inclusive finance on R&D investment of pharmaceutical enterprises may be significantly different due to the size of enterprises. By analyzing the data of listed companies, pharmaceutical manufacturing enterprises are divided into two sub-samples: large enterprises and small and medium-sized enterprises. From the regression results of column (5) in Table 11, it can be seen that the regression coefficient of the digital inclusive financial index is significantly positive under the sample of small and medium-sized enterprises, which indicates that the development of digital inclusive finance can promote the R&D investment of small and medium-sized pharmaceutical enterprises. From the regression results of Column (6) of Table 11, it can be seen that under the sample of large pharmaceutical enterprises, the digital inclusive financial index is significantly positive at the 10% level, which indicates that the development of digital inclusive finance can play a role in promoting large pharmaceutical enterprises. Comparing the results of Column (5) and Column (6) in Table 11, it can be found that under the sample of small and medium-sized enterprises, the regression coefficient of the digital inclusive financial index is larger and the significance level is higher, which indicates that the development of digital inclusive finance has a greater marginal effect on small and medium-sized pharmaceutical enterprises. For large enterprises, they usually have strong market competitiveness and financing ability, and can easily obtain the required funds from traditional financial institutions. Therefore, the development of digital inclusive finance is more of an additional boost for them. These enterprises often have more robust and long-term planning in terms of technological innovation. Digital inclusive finance provides them with more financing channels and choices, allowing them to respond more flexibly to market and technological challenges. However, for small and medium-sized enterprises, the situation is very different. Small and medium-sized enterprises are often difficult to obtain sufficient financing support from traditional financial institutions because of imperfect financial system, weak profitability and high risk. Digital inclusive finance can effectively broaden the financing channels of small and medium-sized enterprises. Through the digital inclusive financial platform, small and medium-sized enterprises can more easily obtain the required funds, thus promoting technological innovation and industrial upgrading.

## 5 Conclusion and recommendations

### 5.1 Conclusion

The modern financial system, empowered by digital technology as an innovative form of in-depth integration of technological revolution and capital services, is continuing to release its catalytic effect on the real economy. Especially in the field of strategic emerging industries such as biomedicine, this new financial model shows unique value creation ability. This paper matches the prefecture-level city data of digital inclusive finance from 2011 to 2022 with the data of Shanghai and Shenzhen A-share listed pharmaceutical manufacturing enterprises and selects the high-dimensional fixed effect model for empirical analysis. The study found that, first, the development of digital inclusive finance has a significant role in promoting the R&D investment of pharmaceutical manufacturing enterprises. The results remain significant after considering endogeneity issues. Its coverage breadth, depth of use, and digital support services degree have played a positive role in promoting. Second, based on the moderating effect model, government subsidies are found to have a moderating effect on the impact of digital inclusive finance on corporate R&D investment. Third, through the subsample regression, the impact of digital inclusive finance on R&D investment of enterprises is heterogeneous in terms of the nature of equity, enterprise size, and regional attributes. With the different nature of equity, digital inclusive finance promotes R&D investment in private enterprises more significantly than in state-owned enterprises. In terms of enterprise size, the impact of digital inclusive finance on R&D investment in small and medium-sized enterprises is greater than that of large enterprises. In terms of the level of traditional financial development in the region where the enterprise is located, digital inclusive finance has a more significant effect on the promotion of R&D investment in pharmaceutical enterprises in regions with a lower level of traditional financial development.

### 5.2 Recommendations

Based on the development of digital inclusive finance and the current status of innovation investment of pharmaceutical listed companies in China, this paper makes suggestions from three levels: government, financial institutions, and enterprises.

At the government level, the government needs to focus on promoting the construction of a digital technology innovation and application system. Through government guidance, it should narrow the differences in regional digitalization processes, focus on strengthening the construction of basic service networks in less developed regions, and give priority to guaranteeing the balanced layout of new types of infrastructure such as telemedicine systems and intelligent supply chain platforms. At the same time, the government should improve the promotion of relevant support policies to provide institutional guarantees for enterprise development. Firstly, strengthen the incentive mechanism for R&D, and implement targeted support for knowledge-intensive industries such as biomedicine and other knowledge-intensive industries, such as additional deductions for R&D expenses and special subsidies to alleviate the pressure of R&D investment by enterprises. Secondly, it should build a closed loop of intellectual property protection, improve the data protection system for new drugs, enhance the rate of return on innovation through measures such as patent term compensation and market exclusivity period setting, and optimize the review standards for generic drugs in order to guide industrial upgrading. In addition, regulators need to establish a dynamic monitoring mechanism for digital finance to prevent the risk of disorderly capital expansion.

At the level of financial institutions, the digital transformation of financial institutions should be promoted, a cloud database of pharmaceutical manufacturing enterprises should be established, and more favorable financial support should be given to enterprises with good credit. Financial institutions should actively promote digital reform, take the initiative to integrate artificial intelligence, cloud computing, blockchain, and other cutting-edge technologies, break the constraints of the traditional financial model, and promote the internal upgrading and innovation of financial institutions. Carve a more accurate corporate credit portrait and promote the diversification of financial services through digital technological means. Encourage the establishment of stable information exchange channels through digital financial means to strengthen close cooperation between banks and enterprises. Construct a cloud database covering government regulators, all financial institutions, and business entities in the region to break the last barrier of data circulation channels. It can be further subdivided under the main database to set up a special sub-database for the pharmaceutical manufacturing industry so as to achieve fine management and in-depth analysis of data.

At the corporate level, companies need to pay attention to cultivating their own research teams, as well as strengthening cooperation with universities and research institutes, and using digital financial tools to enhance R&D. Companies need to frequently understand the news of the financial market, grasp the latest government support policies, get timely access to industry dynamics, and eliminate the problem of poor information. Pharmaceutical manufacturing companies, in particular, need to understand the key role of science and technology innovation in the survival and development of enterprises. We should focus on building a professional R&D team, attracting technical talents by improving remuneration packages, and enhancing the overall R&D capability. Enterprises that do not have independent R&D capabilities for the time being can take the initiative to contact scientific research organizations for cooperation and break through geographical constraints with the help of new technologies such as remote collaboration to initiate innovative R&D projects. Enterprises should also continue to explore new uses of digital finance to enhance R&D efficiency through more application scenarios. By the smooth development of individual enterprises to drive the progress and upgrading of the entire pharmaceutical manufacturing industry in China.

## 6 Shortcomings and prospects

There are still many shortcomings in this study, which may also be worth further exploration. Firstly, this paper selects the ratio of R&D investment to operating income to measure the level of enterprise R&D investment, due to the fact that China’s enterprise patent data is not easy to obtain, and the credibility is not high, so it does not measure the innovation level of the enterprise from the aspect of output, and it is only researched from the perspective of enterprise innovation input. Secondly, some data, such as per capita GDP of prefecture-level cities and business income data of enterprises, have more missing values in the database, so there may be some errors by looking up statistical yearbooks and annual reports of companies and enterprises. Third, this paper analyzes the correlation only in terms of the relationship between digital financial inclusion, government subsidies, and firms’ R&D investment, but in practice, there may be other unobserved factors (e.g., technology diffusion, risk pricing, policy incentives, etc.) affecting firms’ innovation. Therefore, a more in-depth analysis of the correlation is necessary in the future.

## Data Availability

The datasets presented in this study can be found in online repositories. The names of the repository/repositories and accession number(s) can be found in the article/supplementary material.

## Footnotes

^1^ https://data.csmar.com/

^2^ http://www.cninfo.com.cn/
